# A Comparison of Diagnostic and Immunohistochemical Workup and Literature Review Capabilities of Online Artificial Intelligence Assistance Models in Pathology

**DOI:** 10.7759/cureus.61075

**Published:** 2024-05-25

**Authors:** Johnika Dougan, Netra Patel, Svetoslav Bardarov

**Affiliations:** 1 Pathology and Laboratory Medicine, St. George's University School of Medicine, St. George's, GRD; 2 Pathology and Laboratory Medicine, American University of Antigua, Antigua, USA; 3 Pathology and Laboratory Medicine, Richmond University Medical Center, Staten Island, USA

**Keywords:** computer science, comparison study, ancillary studies, differential diagnoses, surgical pathology, artificial intelligence

## Abstract

Artificial intelligence (AI) is a suite of technologies that enables computers to learn and interpret information like human cognition. It has found applications across various fields, including healthcare, agriculture, astronomy, navigation, and robotics. Within healthcare, AI has the potential to enhance diagnostic accuracy, facilitate drug research, and automate patient experiences. This comparative study focuses on the proficiency of AI in generating accurate differential diagnoses in the field of pathology. Six medical vignettes were crafted, and each scenario was then input into three different AI platforms. The pathologist reviewed and determined the most accurate AI model.

## Introduction

Artificial intelligence (AI) models are computational programs that leverage algorithms to analyze data and make future predictions without human intervention [1]. As a subfield of computer science, AI aims to enable computers to process and interpret data like human cognition. Two key concepts that are crucial to understanding AI operations are machine learning and deep learning.

Deep learning, a subset of machine learning, involves a computer system learning from internally accumulated data over time, serving as the foundation for machine learning [2]. Machine learning, on the other hand, encompasses the steps a computer takes to learn from historical data.

The core principles that underlie deep learning are more akin to biological processes than to traditional computing: interconnecting processing components, or nodes, to interact dynamically like human neurons, thereby creating an artificial neural network [3,4]. There are numerous deep neural network models, among which the convolutional neural network (CNN) is predominantly used [5]. CNN is driving the progress for medical image analysis and plays a crucial role in computer vision, a field that equips machines with the ability to "see" and decode visual data [6].

Machine learning can be categorized into three types: supervised, unsupervised, and semi-supervised. Supervised machine learning involves a human operator inputting information and training the AI model to mimic their thought process. The two most common supervised machine learning algorithms encountered in pathology are classification, where an algorithm predicts a label, and regression, where an algorithm predicts a numerical value [7]. Unsupervised machine learning does not require human input; instead, it relies solely on software to identify patterns for the AI model to emulate. Semi-supervised machine learning involves human and software training [2]. Through their ability to learn and adapt, these AI models can revolutionize various fields, from scientific research to healthcare, by providing novel ways to analyze data and make evidence-based decisions.

In contemporary medicine, artificial intelligence is harnessed across many specialties to enhance patient care. When appropriately utilized, AI has the potential to augment patient outcomes and mitigate human error [8]. For instance, deep learning algorithms have demonstrated remarkable proficiency in diagnosing tuberculosis from chest X-ray images, achieving an accuracy rate of 96% after being trained on hundreds of such images [9].

AI's role in precision medicine is also noteworthy, as it can assist the pathologist's ability to predict the likelihood of success for various therapeutic approaches based on individual patient characteristics and treatment paradigms [10]. In the surgical field, sophisticated robotic systems assist human surgeons or even perform operations autonomously. A procedure exemplifying the use of AI in surgical assistance is the deployment of the da Vinci Robotic Surgical System. This system operates on a "master-slave" principle, where a human surgeon performs all surgical maneuvers within a console setup [11]. These maneuvers are then conveyed to the robotic arms positioned at the patient's surgical site. Similar systems are also utilized in hospitals and laboratories for repetitive tasks, administrative procedures [12], rehabilitation, and physical therapy. Virtual nurse assistants [13] even leverage voice and AI technologies to conduct wellness checks. All these examples show how AI is playing a transformative role in the field of medicine.

This paper will focus on how AI can be used in the field of surgical pathology. One of the most notable impacts of AI in pathology is AI's ability to interpret patient history, symptoms, and histopathology results to suggest a list of potential differential diagnoses. This study will focus on conducting a comparative analysis of three distinct artificial intelligence (AI) assistance models. The primary objective is to evaluate their ability to generate differential diagnoses based on microscopic descriptions, suggest ancillary studies like immunohistochemical stains, revise their differential diagnoses based on the suggested immunohistochemical stains, and provide proper references to peer-reviewed publications and other vetted medical sources. Our small study also aims to assess the efficacy and accuracy of these AI models in interpreting and processing textual information, thereby enhancing the process of disease diagnosis.

## Materials and methods

We compared the diagnostic performance of three leading artificial intelligence (AI) chatbots, YouChat (you.com), ChatGPT4 (openai.com), and Claude (claude.com), across six hypothetical case scenarios representing commonly encountered pathologic entities. YouChat provides personalized AI search capabilities with customizable modes tailored to user needs. ChatGPT4 leverages natural language processing for conversational interactions and in-depth analysis. Claude is an advanced AI system renowned for data analysis, pattern recognition, and machine learning proficiency. Paid subscriptions were obtained to access the full capabilities of each platform.

The case vignettes incorporated pertinent patient demographics, clinical history, symptomatology, and histologic findings. Specific queries tested the chatbots' abilities to provide differential diagnoses, recommend relevant immunohistochemical stains for the correct diagnosis, formulate an extensive differential diagnosis based on these positive immunostains, and identify key review articles. Responses from the three AI models were compiled into a blinded table alongside the intended diagnoses. A board-certified pathologist, unaware of the source models, then evaluated the accuracy of the differential considerations and immunostaining recommendations for each case based on expert knowledge.

In one scenario, a 30-year-old female presented with a thyroid nodule, and a fine needle aspiration (FNA) revealed features consistent with papillary thyroid carcinoma. Another case involved a 65-year-old male diagnosed with chronic lymphocytic leukemia/small lymphocytic lymphoma (CLL/SLL) with a peripheral blood smear showing lymphocytosis and a lymph node biopsy demonstrating a nodular infiltrate of small mature lymphocytes. A third scenario depicted a 66-year-old male with elevated prostate-specific antigen (PSA) levels and biopsy findings indicative of prostatic adenocarcinoma. For a fourth case, a 10-year-old female presented with precocious puberty, and histologic examination of an ovarian mass revealed a juvenile granulosa cell tumor. In the fifth vignette, a 75-year-old male presented with a large soft tissue mass on the thigh, with biopsy demonstrating undifferentiated pleomorphic sarcoma. Lastly, a 30-year-old female presented with episodes of hypoglycemia, and imaging studies revealed a pancreatic mass, ultimately diagnosed as a pancreatic neuroendocrine tumor.

This single-blind study design aimed to eliminate potential biases during the assessment of the AI-generated outputs. The AI models You.com, ChatGpt4, and Claude 3 were anonymized and referred to as Model A, B, and C, respectively. Systematic comparison of the models' performance highlighted their strengths and limitations in synthesizing clinicopathologic information and applying clinical reasoning skills to render diagnostic impressions across a diverse range of pathologic disorders.

## Results

This study evaluated the diagnostic performance of three artificial intelligence (AI) chatbots, ChatGPT4, YouChat, and Claude, across six clinical case scenarios. For each scenario, the intended response denoted the correct diagnosis as determined by the study designers. The results are summarized in Tables 1-6.

**Table 1:** Chronic Lymphocytic Leukemia DX = diagnosis, DDX = differential diagnosis, CD = cluster of differentiation

Case: A 65-year-old male presents with unexplained weight loss for the past two months. He has experienced persistent fatigue in the afternoons and at night. Large, firm lymph nodes are bilaterally in the cervical and supraclavicular regions. Excision of lymph nodes shows a diffusely effaced architecture with no follicular structures. The cells are mature, small lymphocytes with no mitoses and no necrosis.		
Intended response: Chronic lymphocytic leukemia, DDX: Follicular lymphoma, mantle cell lymphoma, marginal zone lymphoma, Hodgkin's lymphoma		
ChatGPT4	YouChat	Claude3
DX: Chronic lymphocytic leukemia	DX: Chronic lymphocytic leukemia	DX: Chronic lymphocytic leukemia
DDX: Chronic lymphocytic, leukemia/small lymphocytic lymphoma, follicular lymphoma, mantle cell lymphoma, marginal zone lymphoma, nonspecific reactive lymphadenopathy	DDX: Hodgkin's lymphoma, non-Hodgkin's lymphoma, metastatic carcinoma, infectious mononucleosis, chronic lymphocytic leukemia	DDX: Chronic lymphocytic leukemia/small lymphocytic lymphoma, follicular lymphoma (grade 1 and 2), mantle cell lymphoma, marginal zone lymphoma (nodal or extranodal), reactive lymphoid hyperplasia
Immunostains: CD5, CD19, CD20, CD23, CD200	Immunostains: CD5, CD19, CD20, CD23, CD200	Immunostains: CD5, CD19, CD20, CD23, CD200
Revised response based on immunostains: Chronic lymphocytic leukemia/small lymphocytic lymphoma, follicular lymphoma, mantle cell lymphoma, marginal zone lymphoma, nonspecific reactive lymphadenopathy	Revised response based on immunostains: Chronic lymphocytic leukemia, mantle cell lymphoma, metastatic carcinoma, infectious mononucleosis, other indolent lymphomas	Revised response based on immunostains: Chronic lymphocytic leukemia/small lymphocytic lymphoma, mantle cell lymphoma, atypical chronic lymphocytic leukemia, CD5+ marginal zone lymphoma, other low-grade B-cell lymphoma

**Table 2:** Papillary Thyroid Carcinoma DX = diagnosis, DDX = differential diagnosis, TTF-1 = thyroid transcription factor 1, CK19 = cytokeratin 19, Tg = thyroglobulin

Case: A 30-year-old female presents with a palpable mass in the midline region of her thyroid isthmus without cervical node involvement. The patient has not experienced any symptoms, but a painless, rigid, fixed, 2 cm nodule was palpated on physical examination. Fine needle aspiration cytology shows cellular specimens composed of clusters of follicular cells with nuclear enlargement, papillary structures with fibrovascular cores, nuclear grooves, nuclear membrane irregularities, chromatin clearing, and nuclear inclusions.		
Intended response: Papillary thyroid carcinoma, DDX: Papillary thyroid carcinoma, follicular thyroid carcinoma, Hashimoto's thyroiditis		
ChatGPT4	YouChat	Claude3
DX: Papillary thyroid carcinoma	DX: Papillary thyroid carcinoma	DX: Papillary thyroid carcinoma
DDX: Follicular thyroid carcinoma, papillary thyroid carcinoma, medullary thyroid carcinoma, Hashimoto's thyroiditis, anaplastic thyroid carcinoma	DDX: Thyroid adenoma, papillary thyroid carcinoma, follicular thyroid carcinoma, Hashimoto's thyroiditis, Hurthle cell adenoma or carcinoma	DDX: Papillary thyroid carcinoma, follicular variant of papillary thyroid carcinoma, follicular thyroid carcinoma, follicular adenoma with papillary hyperplasia, Hashimoto's thyroiditis with papillary hyperplasia
Immunostains: TTF-1, CK19, galectin-3 protein, Tg	Immunostains: TTF-1, CK19, galectin-3 protein, Tg	Immunostains: TTF-1, CK19, galectin-3 protein, Tg
Revised response based on immunostains: Papillary thyroid carcinoma, follicular thyroid carcinoma, medullary thyroid carcinoma, Hashimoto's thyroiditis, anaplastic thyroid carcinoma	Revised response based on immunostains: Papillary thyroid carcinoma, follicular thyroid carcinoma, Hurthle cell adenoma or carcinoma, benign thyroid adenoma, Hashimoto's thyroiditis	Revised response based on immunostains: Papillary thyroid carcinoma, follicular variant of papillary thyroid carcinoma, follicular thyroid carcinoma, medullary thyroid carcinoma, anaplastic thyroid carcinoma

**Table 3:** Prostatic Adenocarcinoma DX = diagnosis, DDX = differential diagnosis, PSA = prostate-specific antigen, PSAP = prostatic acid phosphatase, AMACR = alpha-methylacyl-CoA racemase, SCC = squamous cell carcinoma

Case: A 66-year-old male presents to the office with several months of progressive lower back pain. He describes the pain as a dull, achy pain that mostly bothers him at night. He also complains of difficulty urinating. A digital rectal examination shows prostate enlargement. Transurethral resection of the prostate was performed, and some of the prostate chips showed small, round glandular structures with infiltrating patterns composed of cells with enlarged nuclei, prominent nucleoli, and amphiphilic cytoplasm.		
Intended response: Prostatic adenocarcinoma, DDX: Prostate glandular atrophy, prostatic sclerosing adenosis		
ChatGPT4	YouChat	Claude3
DX: Prostatic adenocarcinoma	DX: High-grade prostate cancer	DX: Prostate cancer
Differentials: Prostatic adenocarcinoma, atypical hyperplasia/prostatic intraepithelial neoplasia, chronic prostatitis, benign prostatic hyperplasia, prostatic intraductal carcinoma	Differentials: Prostate cancer, benign prostatic hyperplasia (BPH), prostatitis, metastatic prostate cancer, other causes of lower back pain	Differentials: Adenocarcinoma of the prostate, benign prostatic hyperplasia, prostatic intraepithelial neoplasia, neuroendocrine tumor (SCC) of the prostate, prostatitis with glandular hyperplasia
Immunostains: PSA, PSAP, homeobox protein NKX3.1, AMACR enzyme	Immunostains: PSA, PSAP, homeobox protein NKX3.1, AMACR enzyme	Immunostains: PSA, PSAP, homeobox protein NKX3.1, AMACR enzyme
Revised response based on immunostains: Prostatic adenocarcinoma, atypical hyperplasia/prostatic intraepithelial neoplasia, chronic prostatitis, benign prostatic hyperplasia, prostatic intraductal carcinoma	Revised response based on immunostains: Prostatic adenocarcinoma, metastatic prostate adenocarcinoma, benign prostatic hyperplasia (BPH), metastatic urothelial carcinoma, other metastatic carcinoma	Revised response based on immunostains: Adenocarcinoma of the prostate, benign prostatic hyperplasia, prostatic intraepithelial neoplasia, neuroendocrine tumor (SCC) of the prostate, atypical small acinar proliferation

**Table 4:** Pancreatic Neuroendocrine Neoplasia DX = diagnosis, DDX = differential diagnosis, Ki-67 = antigen Kiel-67

Case: A 30-year-old female presents with tremors, palpitations, headaches, and nervousness for a few months. Abdominal ultrasound shows a pancreatic tail mass measuring 1.5 cm. A core biopsy of the mass shows monotonous, small, round blue cells with salt and pepper-like chromatin and scant cytoplasm. Mitotic figures or necrosis were not present.		
Intended response: Pancreatic neuroendocrine tumor (insulinoma), DDX: Pheochromocytoma, islet cell hyperplasia (nesidioblastosis)		
ChatGPT4	YouChat	Claude3
DX: Pheochromocytoma	DX: Pheochromocytoma	DX: Pheochromocytoma
Differentials: Pancreatic neuroendocrine tumor, insulinoma, pheochromocytoma, gangliocytic paraganglioma, solid pseudopapillary neoplasm	Differentials: Pancreatic neuroendocrine tumor, pancreatic pseudocyst, metastatic tumor, pancreatic adenocarcinoma, benign pancreatic lesion	Differentials: Insulinoma, neuroendocrine tumors, solid pseudopapillary neoplasm, pancreatic metastasis, lymphoma
Immunostains: Chromogranin A, synaptophysin, Ki-67	Immunostains: Chromogranin A, synaptophysin, Ki-67	Immunostains: Chromogranin A, synaptophysin, Ki-67
Revised response based on immunostains: Pancreatic neuroendocrine tumor, insulinoma, pheochromocytoma, gangliocytic paraganglioma, solid pseudopapillary neoplasm	Revised response based on immunostains: Pancreatic neuroendocrine tumor, pancreatic pseudocyst, metastatic tumor, pancreatic adenocarcinoma, benign pancreatic lesion	Revised response based on immunostains: Insulinoma, neuroendocrine tumors, solid pseudopapillary neoplasm, pancreatic adenocarcinoma, lymphoma

**Table 5:** Juvenile Granulosa Cell Tumor DX = diagnosis, DDX = differential diagnosis, CD = cluster of differentiation, MIC2 = microneme protein 2

Case: A 10-year-old child presents with breast tenderness and enlargement that has progressed over the past seven months. She appears more developed than her current age. Abdominal ultrasound shows an enlarged left ovary. The patient underwent a left oophorectomy, which demonstrates effaced nodular architecture at low power. The tumor cells are luteinized with round hyperchromatic nuclei with small nucleoli. The nuclear contour is irregular, and there is a high mitotic rate. Marked pleomorphism is not present.		
Intended response: Juvenile granulosa cell tumor, DDX: Teratoma, yolk sac tumor, embryonal carcinoma		
ChatGPT4	YouChat	Claude3
DX: Precocious puberty	DX: Juvenile granulosa cell tumor	DX: Juvenile granulosa cell tumor
DDX: Juvenile granulosa cell tumor, thecoma, hormone-secreting ovarian teratoma, Sertoli-Leydig cell tumor, ovarian adenoma, adenocarcinoma	DDX: Juvenile granulosa cell tumor, Sertoli-Leydig cell tumor, gonadoblastoma, yolk sac tumor, immature teratoma	DDX: Juvenile granulosa cell tumor, Sertoli-Leydig cell tumor, dysgerminoma, yolk sac tumor, immature teratoma
Immunostains: Alpha-inhibin, calretinin, vimentin, CD99 (MIC2), S100 protein, cytokeratin	Immunostains: Alpha-inhibin, calretinin, vimentin, CD99 (MIC2), S100 protein, cytokeratin	Immunostains: Alpha-inhibin, calretinin, vimentin, CD99 (MIC2), S100 protein, cytokeratin
Revised response based on immunostains: Juvenile granulosa cell tumor, Sertoli-Leydig cell tumor, thecoma, granular cell tumor, ovarian teratoma	Revised response based on immunostains: Juvenile granulosa cell tumor, Sertoli-Leydig cell tumor, gonadoblastoma, yolk sac tumor, immature teratoma	Revised response based on immunostains: Juvenile granulosa cell tumor, Sertoli-Leydig cell tumor, gonadoblastoma, yolk sac tumor, immature teratoma

**Table 6:** Undifferentiated Pleomorphic Sarcoma DX = diagnosis, DDX = differential diagnosis, CD = cluster of differentiation, Ki-67 = antigen Kiel-67

Case: A 75-year-old male with retroperitoneal mass and liver lesion. Histology shows a spindle cell neoplasm with necrosis, mitoses, and nuclear atypia.		
Intended response: Undifferentiated pleomorphic sarcoma, DDX: Liposarcoma, gastrointestinal stromal cell tumor, angiosarcoma		
ChatGPT4	YouChat	Claude3
DX: Leiomyosarcoma	DX: Sarcoma	DX: Retroperitoneal/metastatic leiomyosarcoma
DDX: Retroperitoneal leiomyosarcoma, dedifferentiated liposarcoma, epithelioid endothelial cell tumor, extragastrointestinal stromal tumor, cystic mesothelioma	DDX: Retroperitoneal leiomyosarcoma, dedifferentiated liposarcoma, epithelioid endothelial cell tumor, extragastrointestinal stromal tumor, cystic mesothelioma	DDX: Leiomyosarcoma, malignant peripheral nerve sheath tumor, undifferentiated pleomorphic sarcoma, dedifferentiated liposarcoma, gastrointestinal stromal tumor
Immunostains: Vimentin, CD163, Ki-67	Immunostains: Vimentin, CD163, Ki-67	Immunostains: Vimentin, CD163, Ki-67
Revised response based on immunostains: Undifferentiated pleomorphic sarcoma, leiomyosarcoma, malignant peripheral nerve sheath tumor, fibrosarcoma, synovial sarcoma	Revised response based on immunostains: Undifferentiated pleomorphic sarcoma, leiomyosarcoma, epithelioid sarcoma, gastrointestinal stromal tumor, synovial sarcoma	Revised response based on immunostains: Undifferentiated pleomorphic sarcoma, leiomyosarcoma, malignant peripheral nerve sheath tumor, gastrointestinal stromal tumor, sarcomatoid carcinoma

In our systematic comparative evaluation, all three artificial intelligence (AI) chatbots, YouChat, ChatGPT4, and Claude, demonstrated proficient diagnostic capabilities across the spectrum of presented pathologic case vignettes. When supplied with relevant clinicopathologic details encompassing patient histories, symptomatology, and histologic findings, each language model accurately rendered the intended diagnosis. This ability to synthesize multimodal data inputs and apply clinical reasoning skills to arrive at cogent diagnostic impressions represents a notable competency of modern AI systems.

Moreover, the chatbots exhibited judicious clinical acumen by consistently recommending appropriate ancillary immunohistochemical studies to further substantiate and refine the initial diagnostic formulations. Upon incorporation of the immunostain results, which altered the clinicopathologic context, the AI engines adeptly revised their impressions in accordance with the new findings. This iterative capacity to reassess and modify conclusions based on evolving data highlights the dynamic nature of these models' reasoning processes.

While YouChat and Claude tailored their outputs with pathology-centric language and framing, ChatGPT-4 adopted a more clinically oriented communication style. YouChat further distinguished itself by furnishing a transparent catalog of the information sources queried during its analyses, coupled with clickable reference links to facilitate quick access to relevant literature. In contrast, Claude and ChatGPT4 did not generate citation links, and Claude occasionally provided non-functional links purportedly directing to scholarly articles.

This multifaceted evaluation underscores the growing sophistication and potential utility of AI language models for enhancing diagnostic workflows and clinical decision support systems in pathology. Concomitantly, it illuminates opportunities for continuous expansion of domain-specific knowledge bases, refinement of output contextualization, and development of robust citation practices to uphold scholarly rigor and credibility as AI applications permeate the biomedical landscape.

## Discussion

Our blinded evaluation of the diagnostic outputs from three leading artificial intelligence (AI) language models, ChatGPT4, YouChat, and Claude, revealed YouChat to exhibit superior performance across several key metrics. These included the accuracy of rendered diagnoses, sophistication of response language, pathology-relevant contextualization, and credibility of furnished reference materials. This assessment was conducted by a board-certified pathologist applying rigorous clinical judgment.

Claude garnered recognition for generating nuanced, highly technical responses replete with precise diagnostic terminology. For a case vignette concerning chronic lymphocytic leukemia (CLL), Claude furnished an impressively comprehensive differential diagnosis spanning "chronic lymphocytic leukemia/small lymphocytic lymphoma (CLL/SLL), follicular lymphoma (grade 1 and 2), mantle cell lymphoma, marginal zone lymphoma (nodal or extranodal), reactive lymphoid hyperplasia" (Table 2). While ChatGPT and YouChat also provided plausible differentials, their outputs lacked the same degree of granular pathologic nomenclature.

In contrast, ChatGPT4 adopted a more clinically oriented communication style, exemplified by its top diagnostic impression of "precocious puberty" for a case scenario depicting a juvenile granulosa cell tumor, a pathologic diagnosis (Table 5). YouChat emerged as an adept amalgam, rendering accurate pathologic differential diagnoses while dynamically revising its conclusions based on supplemental immunohistochemical data, all while maintaining a sophisticated, pathology-centric vernacular.

A key distinguishing feature of YouChat was its consistent provision of vetted reference citations to substantiate its outputs, in alignment with established evidence-based practices. While ChatGPT and Claude also furnished references, some were unreliable, non-functional links or cited sources that did not corroborate the stated claims upon review.

Our findings highlight the potential value of YouChat as an ancillary diagnostic tool in the field of anatomic pathology. It is important to note that the implementation of AI language models should be accompanied by expert medical oversight to ensure accuracy and minimize any errors or gaps in knowledge that may arise from these rapidly evolving technologies. "AI hallucination" is a term used to describe a situation where AI creates a seemingly credible but entirely fictitious response. OpenAI's records, for example, admit that the responses generated by ChatGPT may seem believable, but they could be nonsensical or incorrect [14,15]. Through the enhancement of training inputs with an array of precise and contextually appropriate data sets and continual training model refinement, we may discover solutions to these prevailing challenges [15].

To uphold transparency and trustworthiness, the AI engines must provide links to relevant peer-reviewed articles and vetted websites that were used to gather information. This allows users to verify the sources and access additional context. We believe that continued collaboration among various disciplines is crucial for optimizing AI's role in improving the quality and efficiency of diagnostic medicine while prioritizing patient safety.

## Conclusions

In conclusion, our comparative assessment revealed YouChat to exhibit superior performance metrics relative to ChatGPT4 and Claude in the diagnostic pathology domain. YouChat consistently provided accurate differential diagnostic considerations, which were revised appropriately upon integration of supplemental immunohistochemical data. Moreover, it substantiated its outputs by furnishing credible scholarly references, thereby enhancing transparency, and aligning with established practices of evidence-based diagnosis. YouChat's ability to contextualize its responses through a pathology-centric lens while maintaining access to a vast knowledge repository emulated the modus operandi of surgical pathologists who synthesize diverse data streams during the morphologic assessment of biologic specimens. This capacity to dynamically tailor communication style based on user needs highlights YouChat's potential for broader clinical implementation as a diagnostic adjunct across healthcare sectors.

However, it is paramount to emphasize that while artificial intelligence chatbots demonstrate increasing sophistication, they should be implemented judiciously under the guidance of domain experts to mitigate potential inaccuracies or knowledge gaps. These models should be viewed as supplementary decision support tools rather than autonomous replacements for professional medical judgment and expertise. Continued multidisciplinary collaboration between clinicians, scientists, and technologists will be crucial to iteratively refine AI systems and delineate their optimal role in enhancing patient care delivery through optimized diagnostic workflows and clinical decision-making.
